# Navigating the Airway Maze: Challenges and Strategies in Deep Neck Space Infections

**DOI:** 10.7759/cureus.72337

**Published:** 2024-10-24

**Authors:** Meena Nandini R, Hariprasad R, Vijayakumar M, Dharshana R, Safana A, Hari Meyyappan M

**Affiliations:** 1 Otolaryngology - Head and Neck Surgery, Kauvery Medical Care (KMC) Speciality Hospitals (I) Ltd., Trichy, IND; 2 Otolaryngology - Head and Neck Surgery, Trichy SRM Medical College Hospital and Research Centre, Trichy, IND

**Keywords:** airway management, deep neck space infections, diabetes mellitus, multidisciplinary approach, nodal origin, primary cause

## Abstract

Abstract

This study is intended to analyze the clinical profile and outcomes of the patients diagnosed with deep neck space infections. Deep neck space infections (DNSI) are commonly caused by nodal and odontogenic origins. Here in our study, we stress the need for appropriate investigations, early surgical intervention, and skillful airway management for the successful outcome of deep neck space infections. Effective management of deep neck space infections includes deep neck space exploration and treating the primary cause, such as dental extraction, removal of foreign body, and mastoidectomy. We highlight the importance of addressing the primary cause; failure to do so can lead to recurrent or persistent infection, which increases morbidity and mortality. This spectrum of disease presents a clinical challenge for both otorhinolaryngologists and anaesthesiologists because of compromised airways.

Methods

In this retrospective study, we evaluated 20 patients affected by deep neck space infections presented to the Otorhinolaryngology department, KMC Speciality Hospitals (I) Ltd., Trichy, Tamil Nadu, India, from May 2023 to April 2024 and managed them by deep neck space exploration.

Results

In our study, 80% were males and 20% were females. 30% of the patients were within the age group of 50-60 years. The common risk factor in our study was diabetes. Regarding the aetiology, 45% of the cases were of nodal origin, whereas 30% were odontogenic infections. Submandibular space is most commonly involved (25%), followed by parapharyngeal and multiple space infections (20%). About 30% of the patients had Streptococcus species as the causative organism in the culture and sensitivity report. Out of 20 patients, four required airway support, out of which an emergency tracheostomy had to be performed for three patients, and one patient was managed by endotracheal intubation at the time of presentation. All 20 patients had to undergo surgery, out of which 19 patients recovered successfully. One patient experienced a fatal outcome secondary to sepsis.

## Introduction

The neck has superficial and deep fascial planes, which encase the structures of the neck. Deep neck space infections (DNSI) include the deeper neck tissues, which are surrounded by multiple layers of deep cervical fascia with potential spaces between them. The deep spaces of the neck can be divided into lateral pharyngeal (parapharyngeal), retropharyngeal, and peritonsillar spaces. The spaces limited to above the hyoid include the submandibular space, the parapharyngeal space, the peritonsillar space, the masticator space, the temporal space, and the parotid space. The only potential space limited to below the hyoid is the anterior visceral space [1].

According to Loperfido et al., the most common cause of deep neck space infection is odontogenic, accounting for 35-42% in their literature [2]. Other causes include pharyngotonsillar infections, penetrating foreign bodies, trauma to the head and neck, suppurative lymphadenitis and salivary gland infections [3,4].

The most common signs of DNSI are trismus, neck swelling, hoarseness, stridor, posterior pharyngeal wall bulge, cervical emphysema, and torticollis. Among those signs, the most important sign to be noticed is stridor; hence, airway management is important. Initially, the airway should be assessed with rigid video laryngoscopy or flexible fibro optic nasoendoscopy [2], whether the patient requires emergency airway management or is capable of maintaining their airway. Maintaining a safe and secure airway remains the most important immediate therapeutic goal. Awake fiberoptic intubation or tracheostomy should be considered [5].

These patients should be investigated with routine blood investigations, microbial culture (swabs) from pustulating skin fistulae, and infected dental sockets or roots. Contrast-enhanced computed tomography (CECT) is performed if there is no imminent airway compromise or the airway is secured, which is the gold standard. Visceral, retropharyngeal, and prevertebral spaces communicate with mediastinum, so it is necessary to include mediastinum in the CT scan field. Ultrasound imaging has to be done to show a sufficiently liquefied abscess to be drained. An orthopantomogram should be done if a dental infection is suspected.

According to culture and sensitivity, the DNSI individuals should be managed with deep neck space exploration and parenteral antibiotics.

## Materials and methods

A retrospective study was conducted from May 2023 to April 2024 at the Department of Otorhinolaryngology, KMC Speciality Hospitals (I) Ltd., Trichy, Tamil Nadu, India. All these procedures were done after obtaining informed consent from all the patients and in accordance with the ethical standards of our institutional ethical committee. The inclusion criteria of our study were deep neck space-infected patients of all ages and genders, irrespective of their diabetic status. Exclusion criteria were specifically targeted patients with infections secondary to malignancy, those who were treated conservatively, and individuals who were lost to follow-up. This approach aimed to ensure a more homogeneous study group focused on the surgical management of DNSI. A total of 20 patients were identified, consisting of 16 males and four females, with ages ranging from 1 to 70 years. The patients presented with abscesses in the submandibular, submental, retropharyngeal, parapharyngeal, carotid, prevertebral, and posterior triangle cervical spaces.

The evaluated parameters include clinical symptoms, cervical space involvement, aetiology, comorbidities, type of imaging, airway management, abscess drainage method, an isolated pathogen with antibiotic sensitivity, the type of antibiotic administered, and the complications that were studied.

All patients were subjected to airway assessment by using a flexible nasopharyngoscope. All routine basic investigations were performed. Specific investigations, such as CECT neck, were done to diagnose the deep neck space involvement and the extent of the disease. The deep neck space exploration methods were systematically documented, with approaches tailored to the specific neck spaces involved. Surgical procedures were performed using standard aseptic techniques, and care was taken to minimize complications. Microbiological analysis played a vital role in guiding treatment; pathogens isolated from abscess cultures were recorded along with their antibiotic sensitivity profiles. This information informed the choice of empirical antibiotics, which were initially administered and then modified based on culture results to ensure effective treatment. A retrospective, descriptive, and analytical study was conducted on all 20 patients.

The sample size for this study was calculated using the following formula, based on a population (N) of 22, an estimated proportion (p) of 50%, and a desired margin of error (d) of 5%, with a design effect (DEFF) of 1. The final sample size was determined to be 19, with a 95% confidence level. This sample size is deemed sufficient to meet the requirements of the study, ensuring that the results are statistically valid and reliable within the specified margin of error and confidence level. The calculation was performed using the below formula:

$Sample size (n) = [DEFF * Np (1-p)]/[(d2/Z21-&alpha;/2*(N - 1) + p*(1 - p)]$

where n is the sample size; DEFF is the design effect; *N* is the population size; p is the estimated proportion of the population; *q* = 1 - *p*; *d* = desired absolute precision (or) absolute level of precision.

Statistical analysis was performed using SPSS (version 26.0, IBM Corp., Armonk, NY). The continuous variable was expressed as mean and standard deviation. Categorical variables was expressed as frequency and percentage. To run a one sample t test in SPSS, click Analyze > Compare Means > One Sample T Test. P<0.05 was considered as statistically significant.

## Results

Age and sex distribution 

**Figure 1:**
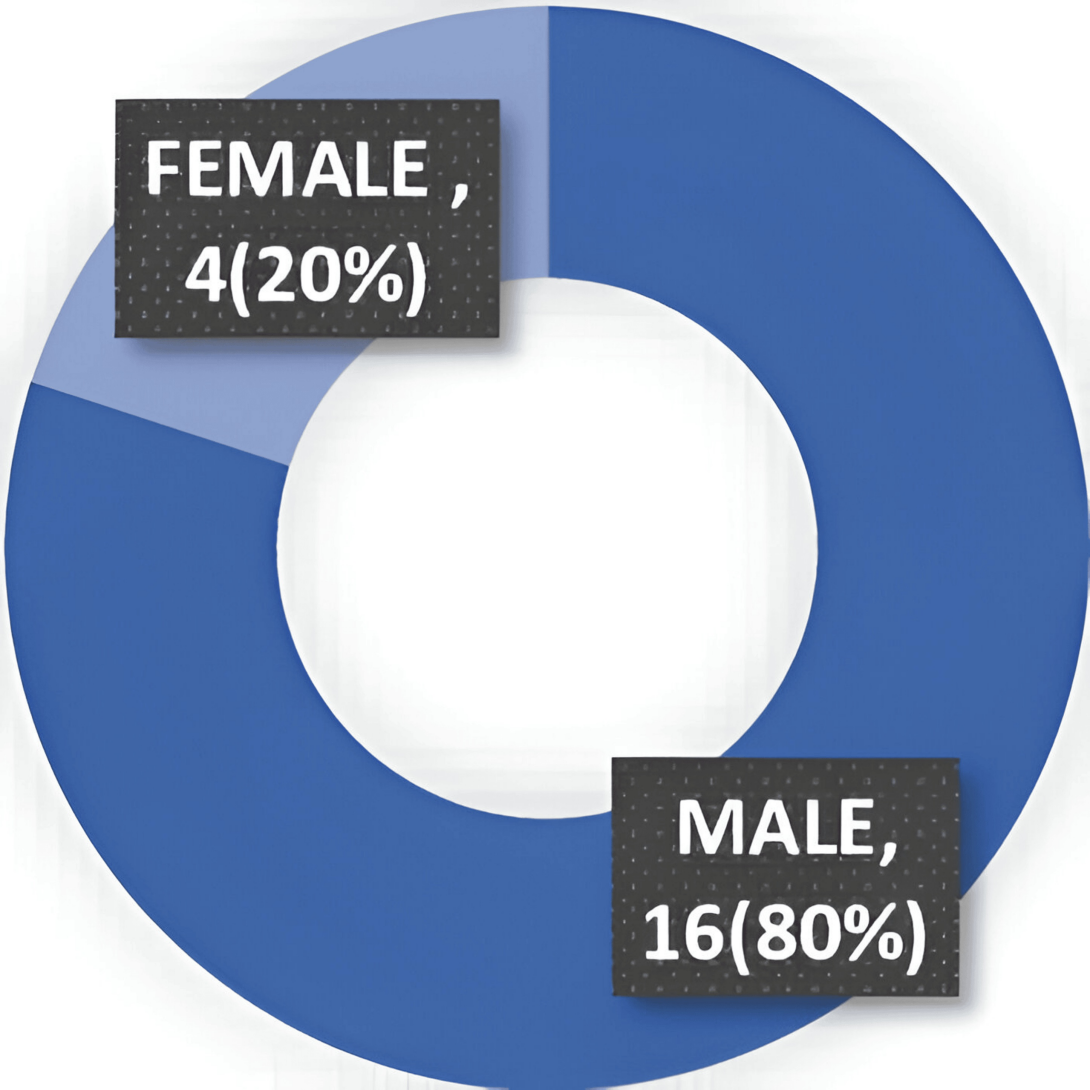
Sex distribution of the patients.

Out of 20 patients, 16 were males (80%) and 4 were females (20%). Figure 1 shows sex distribution.

**Figure 2:**
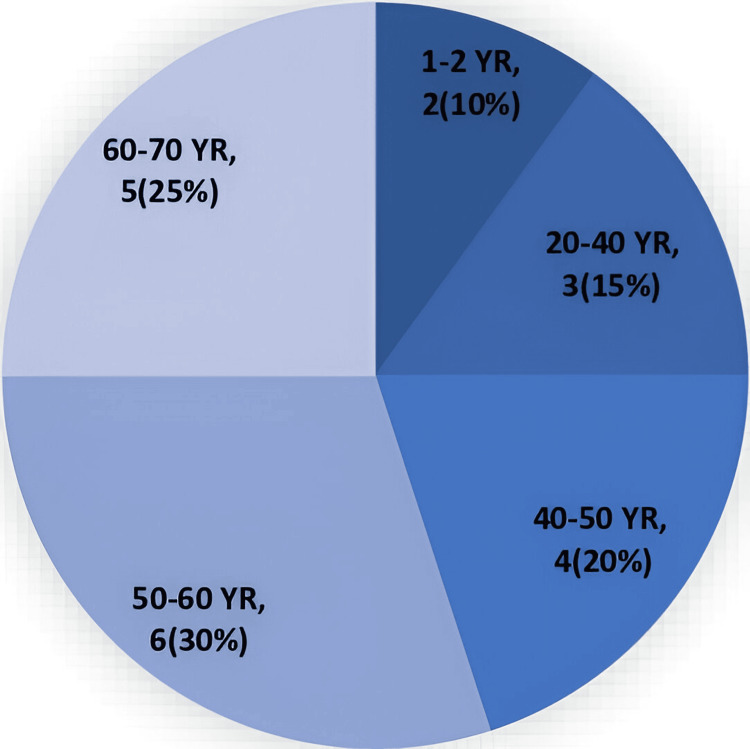
Age distribution of the patients.

Our study included patients with a wide age range, with the youngest being one year old and the oldest being 66 years old. This study included two paediatric cases. Figure 2 shows the age distribution of the patients.

Risk factor

Diabetes was the most common risk factor, found in 12 out of the 20 patients. Among all these patients, the most affected age group was between 60 and 70, with five patients (Table 1).

**Table 1:** The number of patients who had diabetes among different age groups.

Age group (years)	Total number of patients had diabetes	Percentage
20–40	1	5%
40–50	3	15%
50–60	3	15%
60–70	5	25%

Symptoms and signs

The signs and symptoms observed in these patients were listed in Table 2.

**Table 2:** Symptoms and signs of the patients.

	Number of patients	Percentage
Symptoms		
Neck swelling with pain	17	85%
Dysphagia to both solids and liquids	3	15%
Odynophagia	4	20%
Neck pain	2	10%
Dyspnea	2	10%
Fever	3	15%
Signs		
Stridor	4	20%
Pharyngeal wall bulge	3	15%
Crepitus with subcutaneous emphysema	1	5%

Aetiology

Table 3 lists the causes of deep neck space infections in our study. The most common cause was infections secondary to lymphadenitis, followed by odontogenic infections.

**Table 3:** Aetiology of deep neck space infections.

Aetiology	Total number of patients	Percentage
Secondary to nodal origin	9	45%
Odontogenic	6	30%
Post esophagoscopy	2	10%
Post foreign body ingestion	1	5%
Pharyngotonsillar infection	1	5%
Otologic (Bezolds abscess)	1	5%

Airway management

In a series of 20 patients, 4 needed emergency airway intervention due to stridor. One was managed by endotracheal intubation, and the three patients underwent emergency tracheostomy (Table 4).

**Table 4:** Management of deep neck space infection. A: number of patients required airway management; B: approaches of deep neck space exploration; C: primary cause management.

		Number of patients	P-value
A	Airway intervention		
	Emergency airway management required (tracheostomy/intubation)	4 (20%)	0.003 (significant two-tailed)
	Not required	16 (80%)	
B	Deep neck space exploration approach		
	External incision	18	90%
	Intra oral drainage	2	10%
C	Primary cause management		
	Tooth extraction	6	30%
	Mastoidectomy	1	5%
	Foreign body removal	1	5%

Investigations

**Figure 3:**
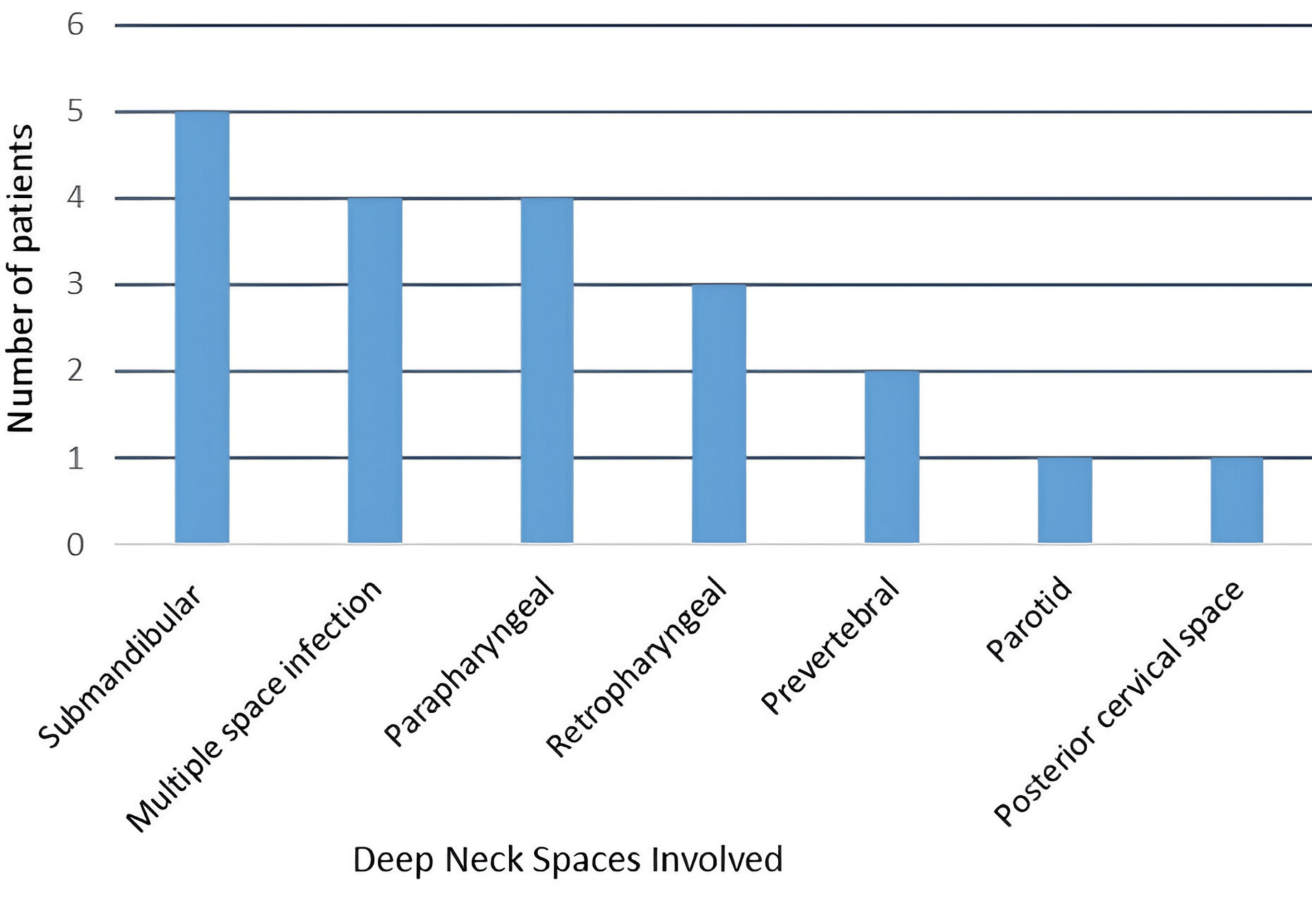
Deep neck spaces involvement.

CECT neck was done in all patients. As shown in Figure 3, five had submandibular space involvement (25%), four in each parapharyngeal (20%) and multiple space involvement (20%), three in retropharyngeal space (15%), two in prevertebral space (10%), one in each parotid(5%) and posterior cervical space (5%).

Management

**Figure 4:**
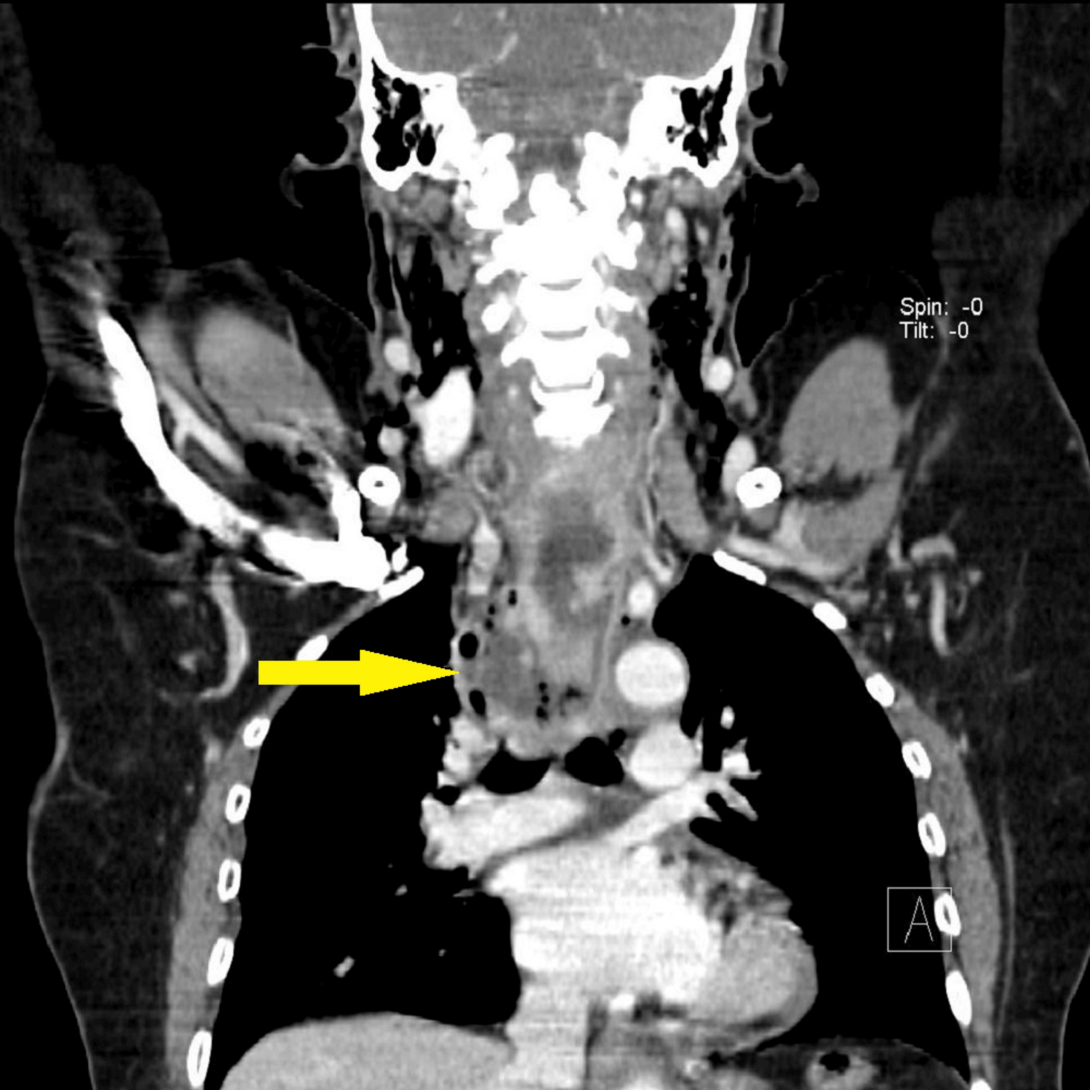
CECT neck (coronal view): showing extensive subcutaneous emphysema on both sides of the neck and anterior chest wall with extensive pneumomediastinum with the large air-fluid level in the posterior mediastinum. Yellow arrow indicating air fluid level in posterior mediastinum.

**Figure 5:**
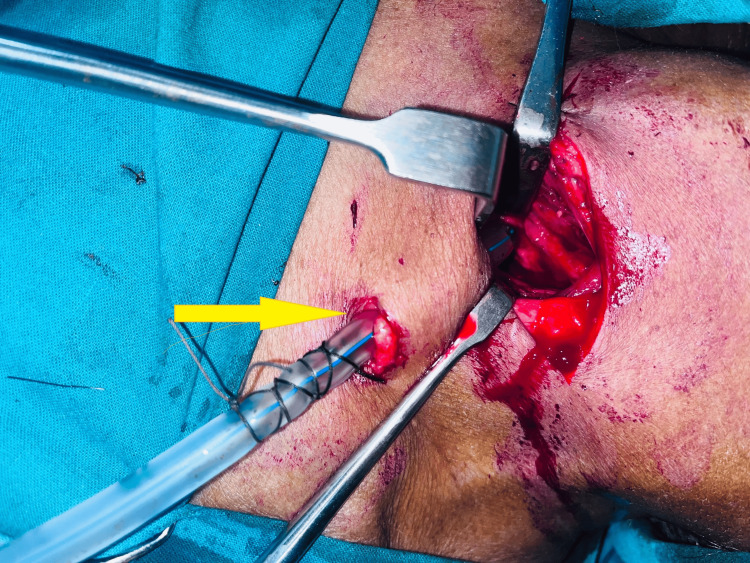
Showing deep neck space exploration with mediastinal drainage.

In this series of 20 patients, all were subjected to deep neck space exploration along with the treatment of primary aetiology. Eighteen patients had deep neck space exploration by external incision. Two patients had intraoral drainage of the abscess (Table 4). Two patients presented with mediastinitis and required mediastinal drainage along with deep neck space exploration (Figures 4-5). Both patients had a history of esophagoscopy for upper oesophageal stricture release.

**Figure 6:**
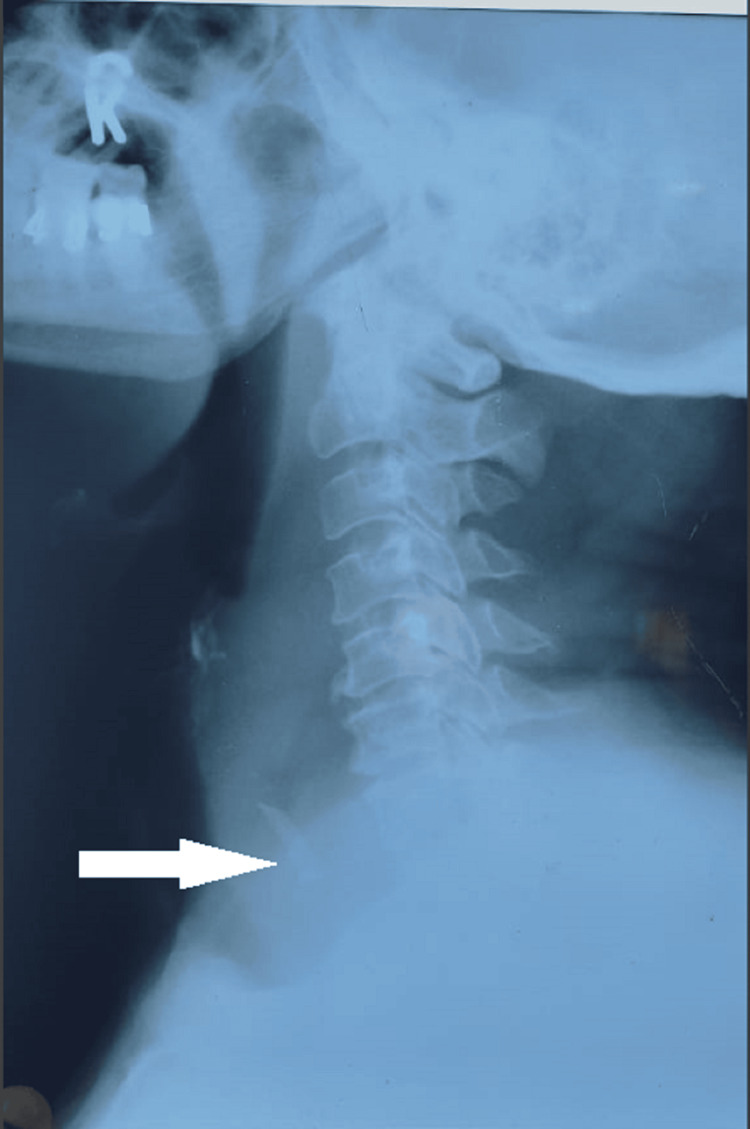
X-ray neck lateral view. White arrow showing foreign body mutton bone in the upper oesophagus with a bulge in the posterior pharyngeal wall.

**Figure 7:**
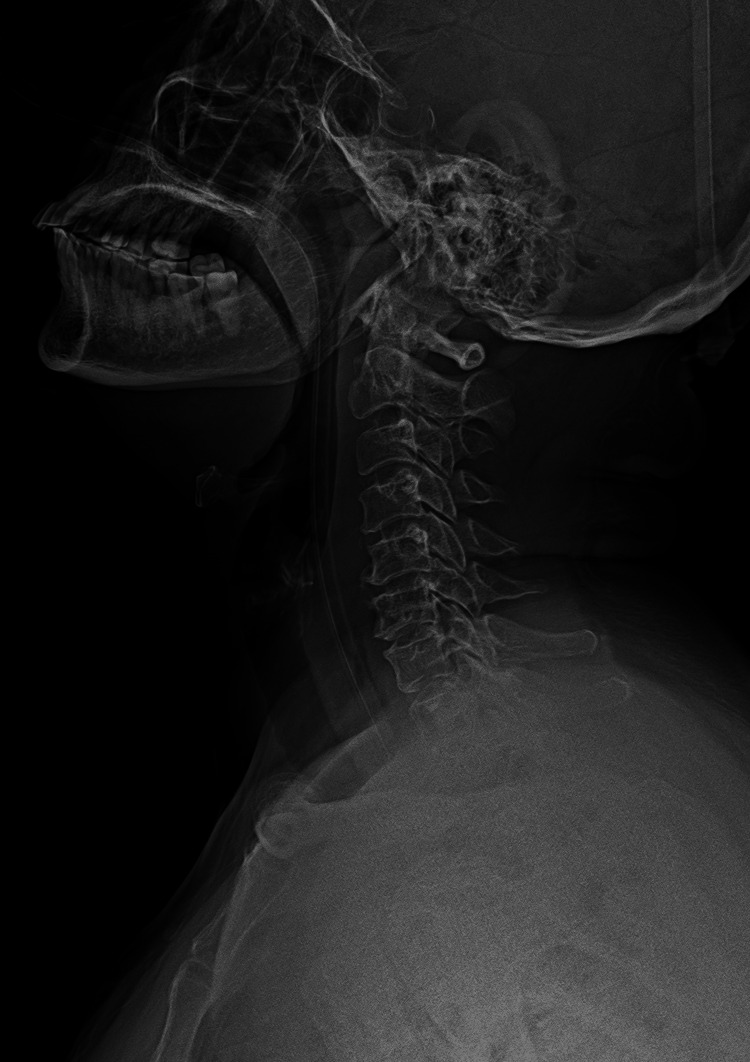
Post-op X-ray neck lateral view (soft tissue), Ryles tube in-situ.

Another patient presented with foreign body impaction in the cricopharynx (mutton bone) with retropharyngeal abscess (Figures 6-7) and required direct laryngoscopic foreign body removal and abscess drainage.

**Figure 8:**
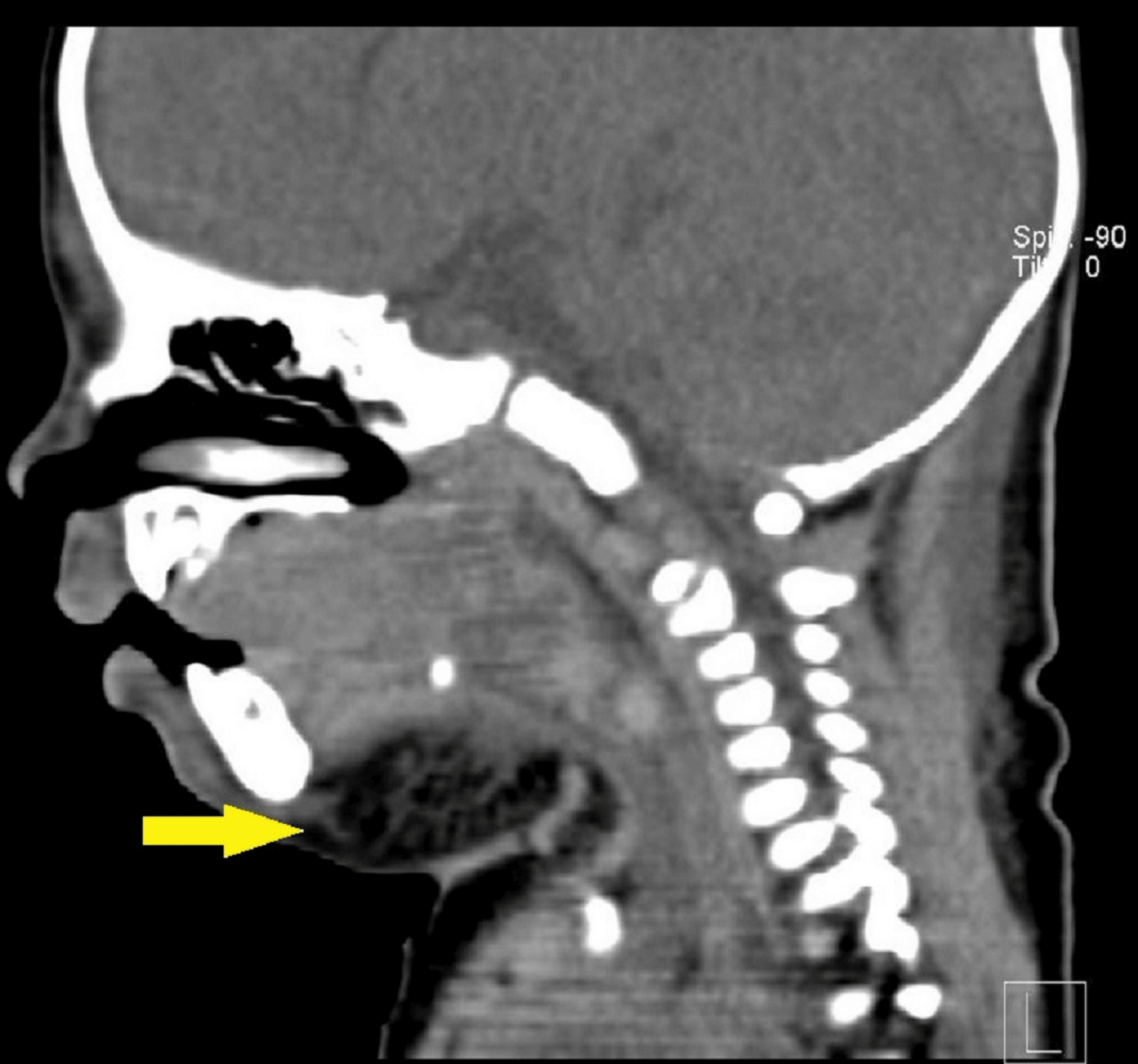
CT neck (sagittal view). Yellow arrow showing submandibular and submental cellulitis.

**Figure 9:**
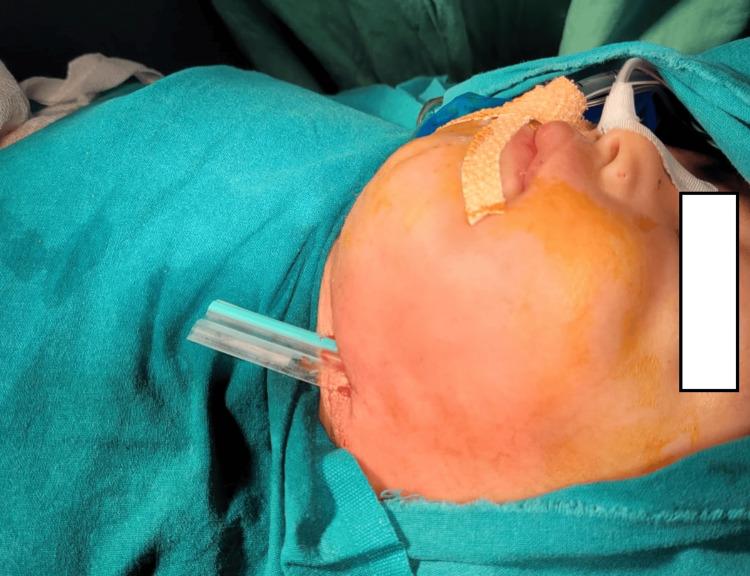
Deep neck space exploration done, with drain in situ.

A one-year-old child underwent deep neck space exploration for submandibular and submental cellulitis. The jugulodigastric node was removed, and exploration revealed Kikuchi lymphadenitis on histopathology (Figures 8-9).

**Figure 10:**
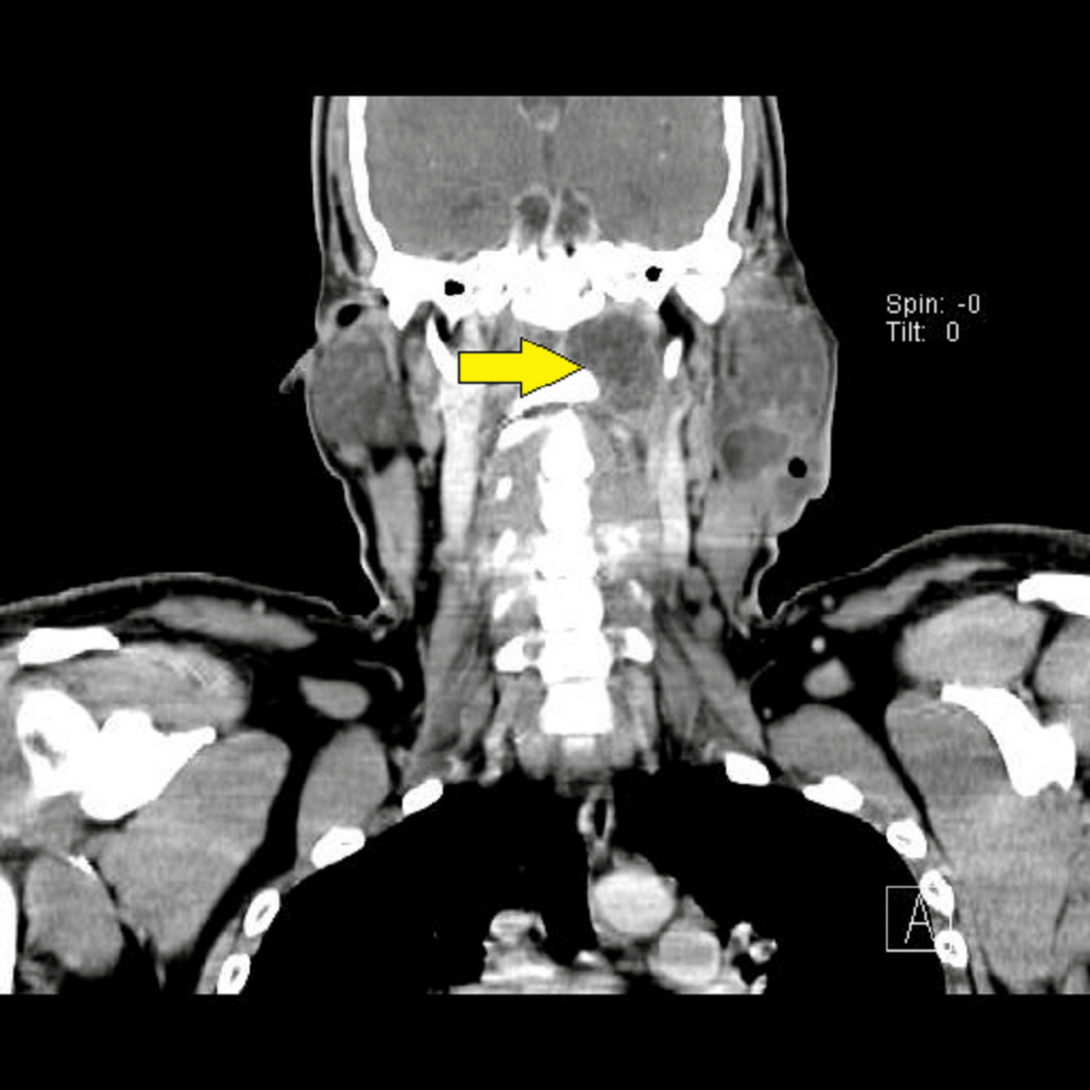
CECT neck (coronal view). Yellow arrow showing left parapharyngeal and parotid abscess.

**Figure 11:**
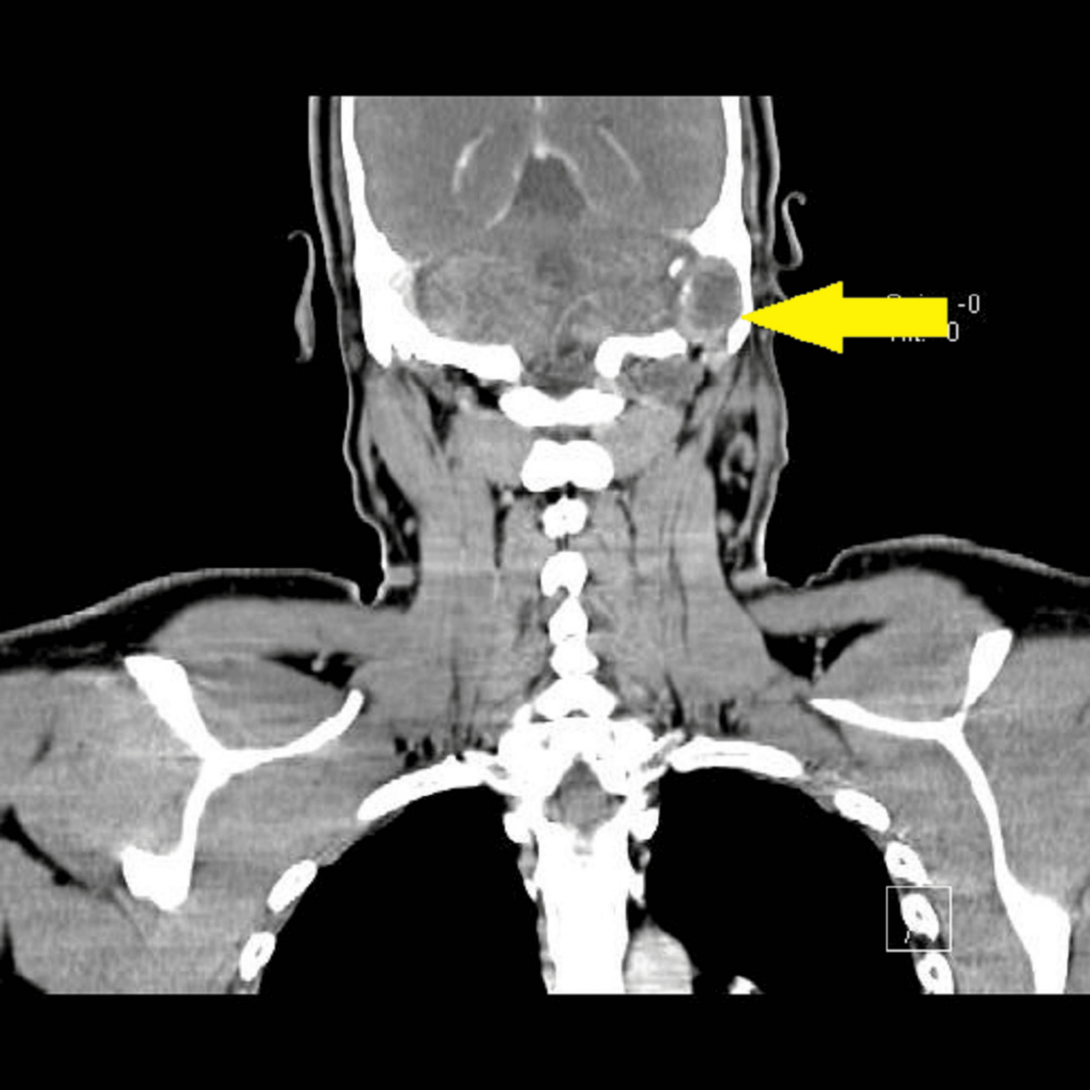
CECT neck (coronal view). Yellow arrow showing opacification in mastoid and erosions in mastoid tip (diagnosed as bezolds abscess leading to parapharyngeal abscess).

**Figure 12:**
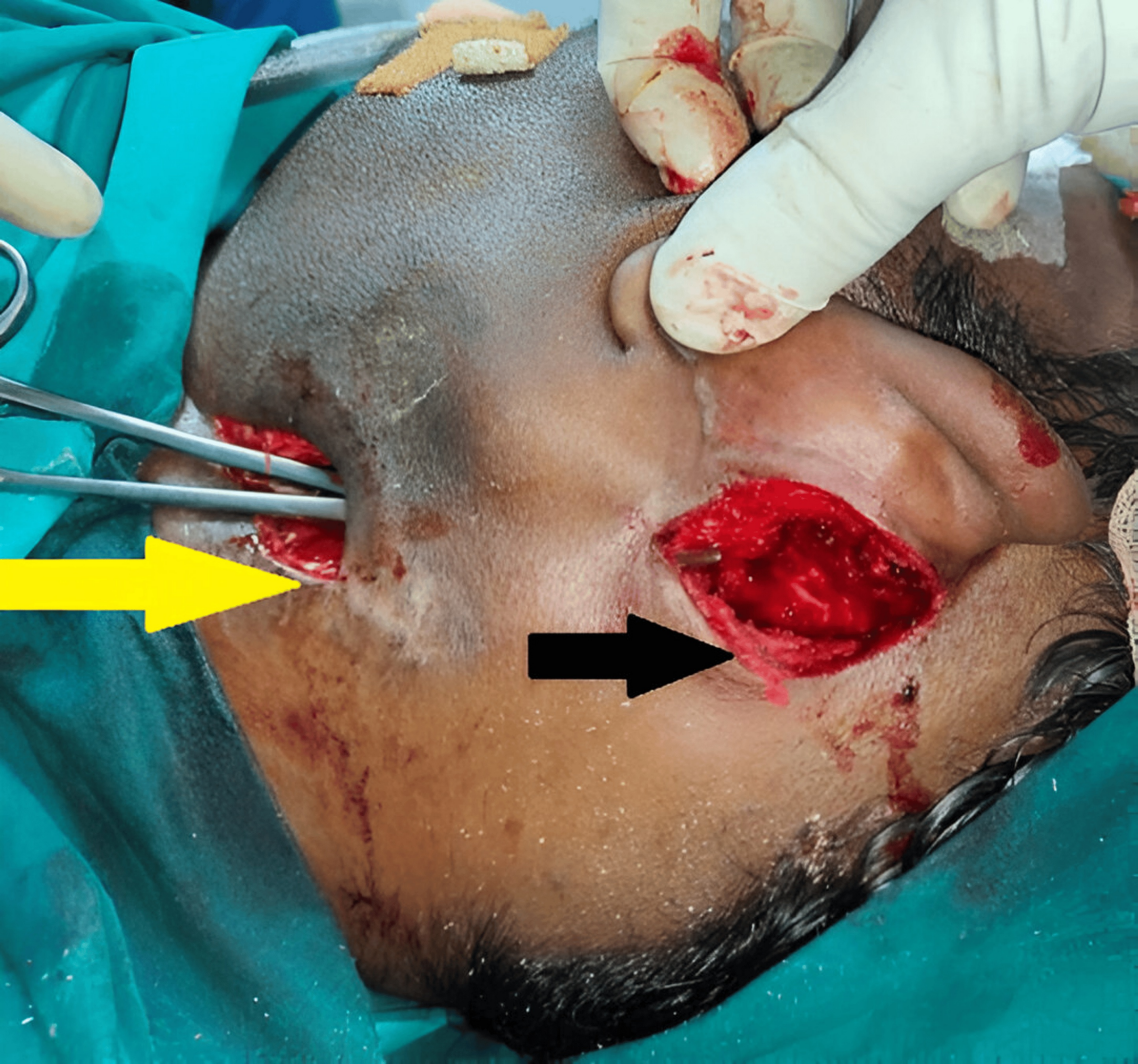
Postaural incision for mastoidectomy and external drainage of abscess. Yellow arrow indicating - neck incision; black arrow indicating - postauricular incision.

**Figure 13:**
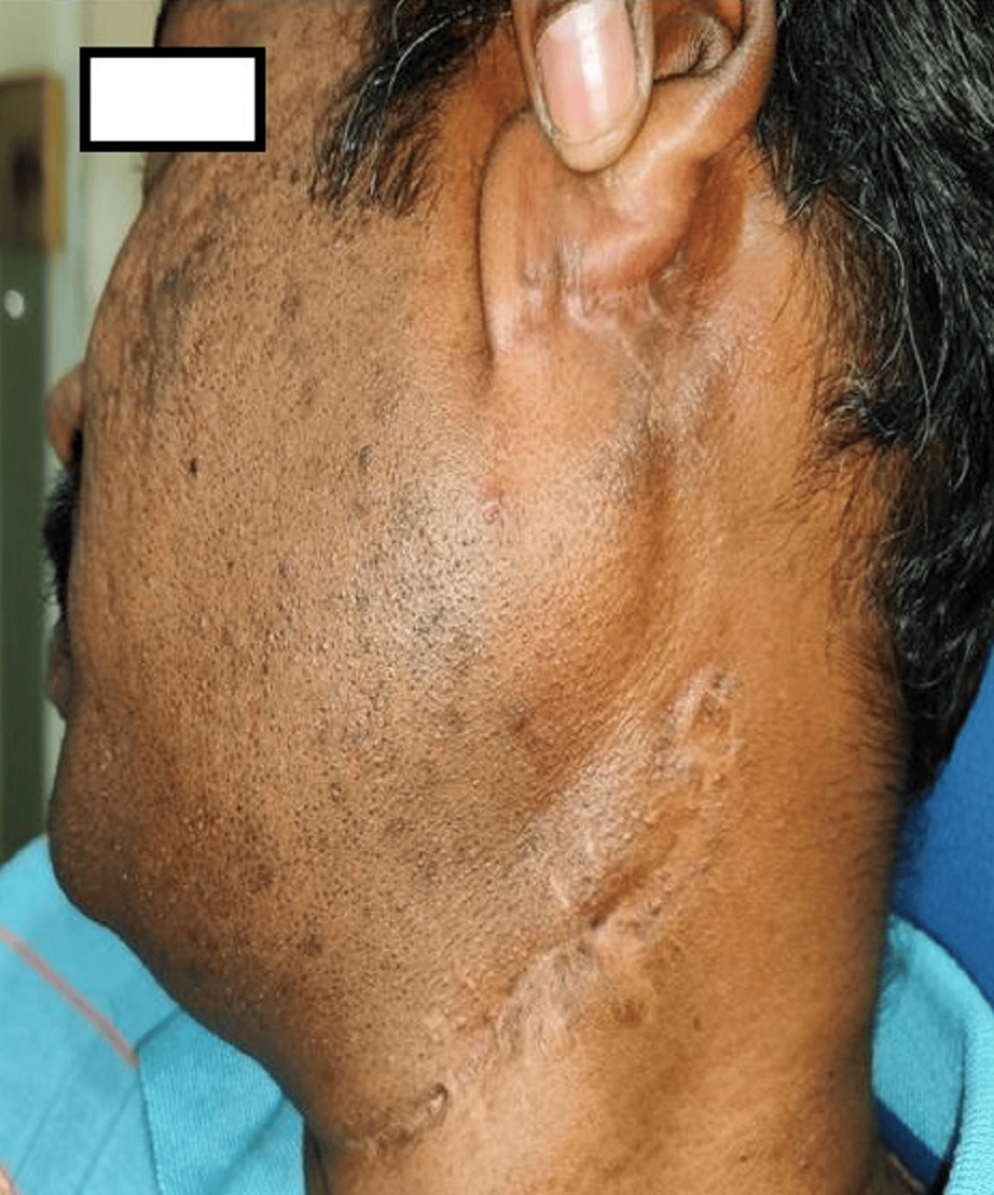
Three months follow up image.

According to Table 4, six patients required tooth extraction, and one patient required mastoidectomy (Figures 10-13).

All patients were started on antibiotics post-surgery according to the culture sensitivity report.

Identified pathogens

**Figure 14:**
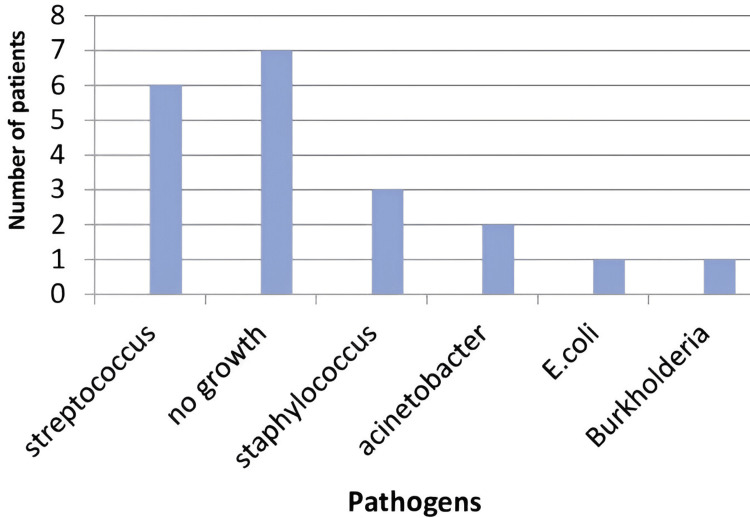
Isolated pathogens.

Out of 20 patients, 13 patients had positive cultures. The most common isolate was Streptococcus species (six out of 13), accounting for 30%, followed by Staphylococcus species (3/13) accounting for 15%, of which two were MSSA and one was MRSA, two out of 13 patients had Acinetobacter species (10%), one had *Escherichia coli*, and one had Burkholderia species (Figure 14).

Complications

Two of the 20 patients experienced mediastinal extension, necessitating mediastinal drainage. One patient presented with multiple deep neck space infections who underwent emergency tracheostomy and deep neck space exploration and succumbed to death on postoperative day 10 in the ICU due to septic shock.

## Discussion

Our study provides insight into the clinical presentation, management, and outcomes of deep neck space infections and highlights prompt recognition and intervention. In our study, males were most commonly affected (80%), which is in concordance with previous studies [2,3,6-8]. However, the reason remains unclear. The most commonly affected age group was 50-60 (30%). The most commonly identified risk factor was diabetes (60%), similar to other studies [6,8]. The statistical significance of this finding is underscored by a p-value of 0.000, suggesting a strong correlation between diabetes and the studied health outcome. This highlights the critical role that diabetes plays in increasing vulnerability to the condition, emphasizing the need for targeted interventions and monitoring for patients with diabetes. Overall, these findings reinforce the importance of addressing diabetes as a critical risk factor in clinical practice and public health initiatives. Huang et al., in their study, confirmed that patients with DNSI associated with diabetes had a longer duration of hospital stay than those without DM [9]. In patients with DM, there is a defect in the host’s immune function, such as cellular immunity complement activation and polymorphonuclear neutrophil function, which increases the risk of vascular complications and episodes of infections [10].

The most common presentation in our study was neck swelling with pain (85%), which is similar to other studies [11,12]. It was followed by odynophagia (20%). Twenty percent of patients presented with stridor and required airway management.

The most common aetiology in our study was infections secondary to nodal origin (45%), followed by odontogenic infections (30%). Other studies have shown that odontogenic infections are more common [6,11,13,14]. Other causes included post-esophagoscopy (10%), foreign bodies (5%), pharyngotonsillar infections (5%), and otologic causes (Bezold's abscess) (5%).

Imaging plays a pivotal role in the evaluation of suspected DNSI, and a CT scan of the head and neck remains the gold standard for the evaluation of DNSI because physical examination alone may misidentify the involved space and the number of involved spaces in 70% of cases [15]. CECT was done in all the patients. The most common space to be involved in our study was submandibular (25%) [11,16], which is in concordance with other studies, followed by parapharyngeal and multiple space infections (20%).

 Our study of 20 patients found that four individuals required emergency airway intervention, highlighting a significant clinical concern. The statistical analysis yielded a p-value of 0.003, indicating a strong association between the need for airway management and the conditions present in this patient cohort. This suggests that a notable proportion of patients may be at risk for severe airway compromise, warranting prompt assessment and intervention. The necessity of airway management underscores the importance of vigilant monitoring and early identification of respiratory distress. Factors such as the underlying medical conditions and the clinical context must be considered, as they may contribute to the risk of airway obstruction. These findings stress the need for healthcare providers to be prepared to act quickly in response to airway emergencies, ensuring that appropriate protocols are in place for rapid evaluation and intervention. Cho et al., in their study, also confirmed that tracheostomy is the gold standard in complex airway management [5].

All patients were managed by deep neck space exploration and treating the primary cause. The purpose of the surgical approach is to remove the triggering cause, drain the purulent collection, and restore airway patency [14]. The external approach was done in 90% of the patients, and intraoral drainage was done in 10%. The external approach predominantly employed a transcervical incision made approximately two fingerbreadths below the angle of the mandible along the skin crease. This positioning intentionally minimizes the risk of injury to the marginal mandibular nerve, a critical consideration during surgical planning. Following the techniques outlined in the literature by Sethi and Stanley [6], exploration of the deep neck spaces was conducted using finger dissection. This method was beneficial for avoiding damage to major vascular structures and preventing residual abscess collections.

Once the deep neck spaces were accessed, a thorough irrigation with metrogyl wash was performed, and a corrugated drain was placed to facilitate drainage. For patients presenting with retropharyngeal abscess, intraoral endoscopic drainage was performed in two cases under orotracheal intubation. The incision for this approach was made at the site of maximal bulging, followed by blunt dissection to release the abscess. The cavity was then visualized endoscopically, allowing for the removal of necrotic debris. Careful haemostasis was secured before extubation to prevent aspiration, and the wound was allowed to heal by secondary intention.

The underlying causes of the infections were addressed through appropriate interventions, such as tooth extractions, foreign body removals, and mastoidectomy. Tooth extractions were carried out in collaboration with the dental team to ensure comprehensive management. Diagnostic laryngoscopy was employed for foreign body removal from the cricopharynx. In one case, a canal wall-down mastoidectomy was performed for a patient presenting with Bezold's abscess, which led to the development of a parapharyngeal abscess. The abscess cavity was meticulously removed from the mastoid, and the mastoid tip cells were exenterated. A drain was placed connecting the mastoid cavity to the external incision made for draining the parapharyngeal abscess, facilitating proper drainage and recovery.

Our study identified Streptococcus species as the most common organism (30%), consistent with other studies [13,17-19].

Ten percent of patients presented with mediastinitis, necessitating mediastinal drainage. Descending necrotizing mediastinitis is the most feared complication; it results from retropharyngeal extension of infection into the posterior mediastinum [12,20].

The mortality rate in our study was 5%, secondary to sepsis. Suehara et al., in their study, showed that the most common cause of mortality was septic shock [12].

Limitations of the study

This was a retrospective study, which could possibly introduce biases and limit the generalizability of our findings. In addition, the duration of our study was only one year, and the single-centre design, which involved a relatively small number of cases, may not accurately represent the broader population. Furthermore, all cases presented with extensive disease stages, precluding early disease presentation and progression assessment. This limitation underscores the need for prospective studies to capture the disease's natural history. Despite these limitations, our study highlights the critical importance of compromised airway management. Future research could benefit from a large multicentric approach and a more extensive patient cohort, which would provide deeper insights into deep neck space infections.

## Conclusions

Deep neck space infections are potentially life-threatening conditions requiring prompt diagnosis and treatment. The complexities of the anatomy of the neck and the variety of pathogens involved pose a challenge in managing these patients. Airway difficulties are a significant concern; early recognition and management are crucial to prevent respiratory compromise. Moreover, the presence of diabetes is a risk factor and can exacerbate the severity of these infections and impact treatment outcomes. Effective management of deep neck space infections requires a multidisciplinary approach, including timely diagnosis, imaging, appropriate antimicrobial therapy, and early surgical intervention.
